# Hemostatic Effect of 20(S)-Panaxadiol by Induced Platelet Aggregation Depending on Calcium Signaling Pathway

**DOI:** 10.1155/2022/8265898

**Published:** 2022-09-20

**Authors:** He Zhang, Yuyao Zhang, Xiaolei Tang, Wenjie Su, Chunhui Yang, Daian Pan, Daqing Zhao, Bin Qi, Xiangyan Li

**Affiliations:** ^1^Research Center of Traditional Chinese Medicine, The Affiliated Hospital to Changchun University of Chinese Medicine, Changchun 130021, China; ^2^Key Laboratory of Active Substances and Biological Mechanisms of Ginseng Efficacy, Ministry of Education, Changchun University of Chinese Medicine, Changchun 130117, China; ^3^Jilin Provincial Key Laboratory of BioMacromolecules of Chinese Medicine, Changchun University of Chinese Medicine, Changchun 130117, China; ^4^Jilin Ginseng Academy, Changchun University of Chinese Medicine, Changchun 130117, China; ^5^College of Pharmacy, Changchun University of Chinese Medicine, Changchun 130117, China; ^6^Department of Tuina, The Affiliated Hospital to Changchun University of Chinese Medicine, 130021, China

## Abstract

*Panax notoginseng* (Burk.) F.H. Chen is the most traditional hemostatic herb in China. Our previous research found that 20(*S*)-protopanaxadiol showed the hemostatic effect. And 20(*S*)-panaxadiol (PD) has a similar structure to 20(*S*)-protopanaxadiol with a dammarane skeleton. So, this article mainly studies the hemostatic effect of PD. The mouse tail amputation and liver scratch models were used to detect the hemostatic effect of PD. Blood routine and plasma coagulation parameters were measured by using a blood analyzer. The platelet aggregometer analyzed the platelet aggregation rate and adenosine triphosphate (ATP) concentration. Moreover, the intracellular calcium concentration ([Ca^2+^]*_i_*), P-selectin (CD62P), PAC-1 (GP IIb/IIIa receptor marker), and cyclic adenosine monophosphate (cAMP) of platelets were also detected. The results showed that PD obviously shortened the bleeding time of the model mouse, affected the RBC and PLT parameters of rats, reduced APTT and TT, elevated FIB concentration, and promoted human/rat-washed platelet aggregation *in vitro*. PD promoted the release of ATP and [Ca^2+^]*_i_* and slightly increased the expression of CD62P and PAC-1 of platelets without 1 mM Ca^2+^. After adding 1 mM Ca^2+^, PD obviously increased ATP releasing and CD62P and GP IIb/IIIa expression rate and decreased the cAMP level of platelets. These parameter changes of PD-caused platelet were inhibited by vorapaxar. Besides, PD increased the phosphorylation of phosphoinositide 3-kinase/protein kinase B/glycogen synthase kinase 3*β* (PI3K/Akt/GSK3*β*) of human platelets. PD is an important hemostatic ingredient in *Panax notoginseng*, which induced platelet aggregation by affecting the calcium signaling and activating the PI3K/Akt/GSK3*β* signaling pathway.

## 1. Introduction

Early bleeding control is essential in metrorrhagia and metrostaxis, wound, and other bleeding disorders. Uncontrolled hemorrhage leads to anemia and even survival-threatening conditions [1, 2]. *Panax notoginseng* (Burk.) F. H. Chen is traditional Chinese medicine, which is the most famous trauma panacea in China [3]. Compendium of materia medica recorded that *Panax notoginseng* could stanch bleeding, disperse blood stasis, relieve pain, and treat most blood diseases. Yu Qiu Yao Jie recorded that *Panax notoginseng* had the effects of heying hemostasis, dredging blood vessels, removing blood stasis, and gathering new blood [4]. In Chinese Pharmacopeia, *Panax notoginseng* possesses the function of promoting blood circulation and removing blood stasis, hemostasis, detumescence, and pain relief. It cures various kinds of bleeding, pain, and swelling. At present, dencichine [5], notoginsenoside Ft1 [6], and PPD [4] reduced the bleeding time of model mice and induced platelet aggregation that showed the hemostatic effect. Despite ongoing endeavors, we think still some hemostatic ingredients are undiscovered.

**Figure 1:**
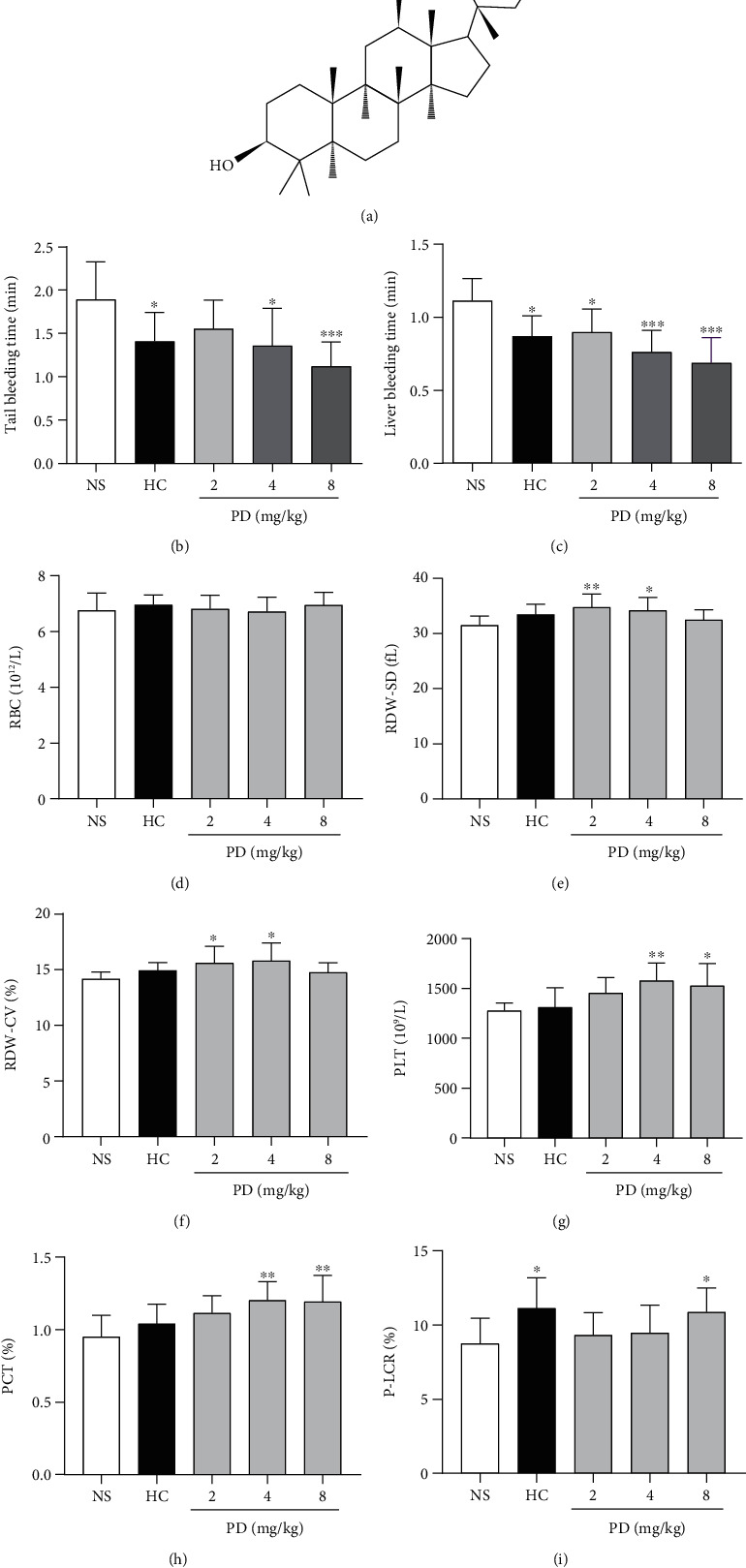
The effect of PD on the mouse bleeding time and the rat blood routine. (a) The chemical structure of 20(S)-panaxadiol (PD). (b) The bleeding time of mouse tail amputation. (c) The bleeding time of mouse liver scratch. (d–f) the effect of PD on red blood cell parameters, including (d) red blood cell (RBC) counts, (e) red cell distribution width-standard deviation (RDW-SD), and (f) red cell distribution width-coefficient of variation (RDW-CV). (g–i) The effect of PD on platelet-related parameters, (g) platelet (PLT) counts, (h) plateletcrit (PCT), and (i) platelet larger cell ratio (P-LCR). The data are expressed as mean ± SD (*n* = 8). ^∗^*p* < 0.05, ^∗∗^*p* < 0.01, and ^∗∗∗^*p* < 0.001 compared to the NS group.

At present, saponins and sapogenins after hydrolysis in the body are important active components in *Panax notoginseng* [7], most saponins have the antithrombosis such as protopanaxadiol-type ginsenoside Rb1 [8], Rg3 [9], Rd, and Rh2 [10], which restrain platelet aggregation and thrombus formation. Protopanaxadiol-type ginsenosides were hydrolyzed by intestinal bacteria, acid, base, or enzymes to yield protopanaxadiol (PPD) [7, 11]. The structure of PPD is unstable, which easily cause changes in C-20 hydroxyl, including protopanaxadiol dehydration, cyclization, and structural transformation and finally generate the dehydrated aglycone (panaxadiol (PD)) [12]. PD is considered a purified sapogenin of diol-type triterpenoid with a dammarane skeleton [13], and its content is 1.92% in *Panax notoginseng* [14]. In recent years, anticancer activities of 20(*S*)-PD (Figure 1(a)) were widely studied [15, 16]. But, until now, few researchers studied the effect of 20(*S*)-PD on the blood system. Our previous research found that PPD could promote the hemostasis of bleeding rats and induce platelet aggregation by depending on calcium signaling [4]. Gao et al.'s research showed that PPD increased the aggregation rate of platelets induced by ADP [10]. Based on the characteristic of the structure with a dammarane skeleton, we speculate that 20(*S*)-PD plays a potential role in platelet aggregation and participates in the hemostasis process.

Hemostasis is an important procedure in hemorrhagic diseases. Platelets, vascular components, and coagulation factors are the main ingredients that participate in the hemostasis process [17]. At first, subendothelial matrixes such as collagen, von Willebrand factor (vWF), and fibronectin are exposed on blood vessel damage; circulating platelets are activated and adhere to the subendothelial surfaces [18]. During this process, platelets change their shape, release a large number of granules, and promote the interaction between injured endothelial cells and platelets [19], which accelerate platelet aggregation and contribute to a series of events in the coagulation cascade leading to thrombin generation and fibrin clot formation that ultimately arrests bleeding [20].

Thrombin formation is initiated by the exposure of tissue factors to plasma coagulation factors after disruption of the vascular endothelium [21]. Thrombin is a key enzyme in the blood coagulation cascade and platelet activator. Protease-activated receptor 1 (PAR1) and PAR4 in human platelet have been demonstrated to participate in most platelet activation by thrombin [22]. The affinity of PAR1-binding thrombin was higher than PAR4, and PAR1 activation leads to a faster and stronger Ca^2+^ into platelets. Ca^2+^ influx in platelets is an important step in activation, shape change, and granules release [23]. PAR1 couples G12/13, Gq, and Gi/z families of G proteins, which activate intracellular signaling pathways of platelet to participate in the coagulation process [24]. Vorapaxar (SCH530348) is a small organic molecule, high affinity, orally active, competitive PAR1 inhibitor [25], which inhibits platelet activation. The latest studies indicate that vorapaxar reduces the risk of myocardial infarction (MI), cardiovascular death, or stroke and increases the risk of moderate or severe bleeding compared with standard of care alone in patients [26, 27].

In our studies, we detected the hemostatic effect of PD *in vitro* and *in vivo*. The results showed that PD shortened the bleeding time of the mouse tail amputation and liver scratch, influenced APTT, TT, and fibrinogen of coagulation parameters, and induced platelet aggregation by regulating calcium signaling and PI3K/Akt/GSK3*β*. PD showed an excellent hemostatic effect. Further studies showed that the hemostatic effect of PD is similar but not identical to thrombin, which is possibly weakly associated with PAR1 on platelets. After adding the vorapaxar, activation, release, and aggregation of platelets induced by PD were reversed. These findings suggest that PD shows the hemostatic effect, will benefit basic science, and aid in the development of effective therapies for hematological disorders.

## 2. Materials and Methods

### 2.1. Materials

20(S)-panaxadiol (PD, purity ≥ 98%) was purchased from Shanghai yuanye BioTechnology (Shanghai, China). Antiplatelet agents such as vorapaxar (VP), ticagrelor (TG), and seratrodast (ST) were purchased from MedChemExpress (New Jersey, USA). The kits for measuring activated partial thromboplastin time (APTT), prothrombin (PT), and fibrinogen were obtained from Nanjing Jiancheng Bioengineering Institute (Nanjing, China). Thrombin time (TT) was purchased from YaJi Biological (Shanghai, China). Hemocoagulase and cyclic adenosine monophosphate (cAMP) ELISA kits were supplied by Jinzhou Ahon Pharmaceutical (Liaoning, China) and Sino Best Biological Technology (Shanghai, China), respectively. FITC-conjugated anti-human CD62P and PAC-1 antibodies were obtained from BioLegend (California, USA). Antibodies against Akt, phospho-Akt (Ser473), PI3K, phospho-PI3K (Tyr607), GSK3*β*, phospho-GSK3*β* (Ser9), and *β*-actin were obtained from Abcam (Cambridge, UK). Platelet function reagents for thrombin (Chrono-Log Corporation, Pennsylvania, USA) and Fluo-3 AM calcium indicators (Beyotime Biotechnology, Shanghai, China) were used in this study.

### 2.2. Animals

Male Wistar rats (200.0 ± 10.0 g) and Kunming mice (20.0 ± 2.0 g) from Liaoning Changsheng Biotechnology Co. Ltd (Liaoning, China, SCXK (Ji)-2016-0003) were used and kept under temperature- (25 ± 1°C), humidity- (60 ± 5%), and 12 h light/dark-controlled conditions allowing *ad libitum* access to food and water. All animal studies were approved by the Institutional Animal Care and Use Committee of the Changchun University of Chinese Medicine (No. 20190133) and performed in accordance with the guiding principles of animal research protocols.

### 2.3. The Measurement of Bleeding Time

According to Yan's and our reports, the bleeding time from different groups was measured in two mice models [4, 28]. 40 male Kunming mice were randomly divided into five groups, including normal saline (NS), hemocoagulase (HC), and PD groups with different doses of 2, 4, and 8 mg/kg PD. After subcutaneous injection of drugs for 4 h, mice were anesthetized *via* intraperitoneal injection (IP) of 4% pentobarbital sodium. In the tail amputation model, mice tails at about 1 cm from the tip were transected with a sterile scalpel and immediately immersed in normal saline at 37°C. The bleeding time from different groups was recorded from the bleeding begin to cessation. In the liver scratch model, the liver bleeding was caused by scratching the lobe at the left lateral position and recorded to calculate the bleeding time. In the end, all mice were euthanized *via* cervical dislocation under anesthesia.

### 2.4. Blood Routine Test

Blood samples from 40 Wister male rats (200.0 ± 10.0 g) of five groups, including NS, HC, and PD groups (2, 4, and 8 mg/kg) were withdrawn from the abdominal aorta and placed in an anticoagulant tube with EDTA to perform routine blood analysis by using the XT-2000i automated hematology analyzer (Sysmex Corporation, Japan), as described previously [4].

### 2.5. Plasma Coagulation Assay

The blood of the aorta abdominal from Wister male rat was withdrawn and then placed in an anticoagulant tube with a 3.8% sodium citrate to obtain the plasma through centrifugation at 3,000 rpm for 15 min. The mixtures with plasma and PD were kept at 37°C for 5-10 min for performing coagulation assays (PT, APTT, TT, and FIB) according to the kit instructions by using an automatic coagulation analyzer (H1201, Jiangsu Horner Medical Instrument Co., Ltd., China). Briefly, after incubation for 10 min at 37°C, 200 *μ*L of the rat plasma mixture with PD (35, 70, or 140 *μ*M) or thrombin (0.5 U/mL) was blended with APTT (200 *μ*L), PT (150 *μ*L), or TT (100 *μ*L) assay reagents to detect APTT, PT, or TT, respectively. The clotting times (s) were recorded immediately and monitored by using an automatic coagulation analyzer. In FIB assay, after incubation for 10 min at 37°C, 200 *μ*L of rat plasma mixture with PD was blended with 100 *μ*L thrombin assay buffer to record clotting time (s) immediately. The standard curve was drawn based on the concentration of fibrinogen (*x*, g/L) and clotting time (*y*, s) (*y* = −0.1505*x* + 57.363) for determining the content of FIB.

### 2.6. Platelet Aggregation, ATP Release, and CD62P/PAC-1 Expression Analyses

Blood samples from humans and rats were collected into 3.8% sodium citrate to prepare human/rat-washed platelets according to the previous report [29]. Briefly, the supernatant of blood was collected after centrifugation (800 rpm × 5 min) to obtain abundant platelets as platelet-rich plasma (PRP), which was washed twice with Tyrode's buffer (pH 7.4) and resuspended to 3 × 10^8^ platelets/mL. 290 *μ*L washed platelets containing 1 mM CaCl_2_ were preincubated with 10 *μ*L PD (17.5, 35, 70, 140, or 280 *μ*M) or thrombin (0.5 U/mL) at 37°C for 5 min to analyze platelet aggregation by a platelet aggregometer with shaking at 1,200 rpm/min (Chrono-Log 700, Pennsylvania, USA). Moreover, the ATP release of platelets incubated with PD (35, 70, and 140 *μ*M) alone or combined with three antiplatelet agents, such as vorapaxar (VP, 10 *μ*M), ticagrelor (TG, 10 *μ*M), or seratrodast (ST, 10 *μ*M), was detected by the luciferin/luciferase reagent. The percentages of CD62P (P-selectin secretion marker, 5 *μ*M) and PAC-1 (activated GP IIb/IIIa receptor marker, 5 *μ*M) in human washed platelets incubated with PD alone or combined with VP were analyzed immediately with a BD FACSAria II flow cytometer (BD Biosciences, California USA). During the analysis, a total of 10,000 events were acquired and repeated at least three separate experiments for analyzing the expression of CD62P or PAC-1 on the surface of platelets. The detailed protocol was shown in the previous report [4]. The human study was approved by the Research Ethics Committee of the Affiliated Hospital to Changchun University of Chinese Medicine (No. CCZYFYLL2017-041). Informed consent was provided for blood donation.

### 2.7. Determination of Intracellular Calcium Concentration

Intracellular calcium concentration, [Ca^2+^]*_i_* was determined with Fluo-3 AM probe, according to the previously reported [30]. Human washed platelets were incubated with Fluo-3 AM (5 *μ*M) at 37°C for 60 min in the dark condition, washed two times, and suspended with Tyrode's buffer for 3 × 10^8^/mL. 0.1 mL PD alone or combined with VP was added to 1.9 mL Fluo-3-loaded platelets, and [Ca^2+^]*_i_* was monitored for 5 min by using a F4500 fluorescence spectrophotometer (Hitachi) at 488 nm and 525 nm to conduct calcium kinetic analysis. According to previously report [31], [Ca^2+^]*_i_* is determined using the equation: [Ca^2+^]_*i*_ = 525 nM × (*F* − *F*_min_)/(*F*_max_ − *F*), where the *F*, *F*_min_ (minimum value), and *F*_max_ (maximum value) represent the fluorescence values and 525 nM represents the Fluo-3 dissociation constant. *F*_min_ and *F*_max_ are minimum and maximum fluorescence value and are measured after the treatment with 10 mM EGTA and 0.1% Triton *X*-100, respectively.

### 2.8. The PAR1 Expression of the Human Platelet

Human washed platelets were incubated with methanol or PD (35, 70, and 140 *μ*M) at 37°C for 10 min, and then the 10 mM EDTA was added to terminate the reaction. After freezing at -80°C and thawing at 37°C for 5 times, the solution was centrifuged at 3,000 rpm for 10 min at 4°C to obtain the supernatants for detecting the concentration of PAR1 using the ELISA kits.

### 2.9. Measurement of cAMP Level

After treating PD (35, 70, and 140 *μ*M) or thrombin (0.5 U/mL) at 37°C for 10 min, the reaction solution of human washed platelets was added the 10 mM EDTA to terminate the reaction, then frozen at -80°C, thawed at 37°C for 5 times, and centrifuged at 3,000 rpm for 10 min to collect the supernatant for cAMP measurement using the ELISA kit. Furthermore, human washed platelets were treated with VP (10 *μ*M) at 37°C for 5 min and then incubated with PD to further investigate the effect of cAMP concentration during platelet aggregation.

### 2.10. Western Blot Analysis

Human washed platelets preincubated with PD alone or combined with VP at 37°C for 15 min were lysed by 100 *μ*L RIPA buffer containing protease/phosphatase inhibitor cocktail (Beyotime Biotechnology). 30 *μ*g proteins were separated by 10% SDS-PAGE gel and transferred to polyvinylidene difluoride membrane for 1 h. After blocking, the specific antibodies against phospho-Akt, Akt, phospho-PI3K, PI3K, phospho-GSK3*β*, GSK3*β*, and *β*-actin were incubated overnight at 4°C, and secondary antibodies were kept at room temperature for 1 h. After washing, the protein band was visualized and analyzed using FluorChem HD2 (California, USA).

### 2.11. Statistical Analysis

All experiment data are shown as the mean ± standard deviations (SD). Statistical significance was determined by one-way ANOVA followed by Dunnett's multiple comparison test by using the GraphPad Prism 8.0 software (GraphPad Inc., California, USA). *p* < 0.05 was considered statistical significance.

## 3. Results

### 3.1. PD Shortened the Bleeding Time of Model Mice and Affected the Parameters of RBC and PLT in Rat

The results of the experiment showed that PD significantly decreased the bleeding time of model mice. In the mouse tail amputation, the bleeding time of mice treated with 4 mg/kg and 8 mg/kg PD was obviously decreased compared to the NS group (*p* < 0.05 or *p* < 0.001, Figure 1(b)). In the liver scratch model, 3 doses of PD significantly shortened the bleeding time of mice compared with the NS group (*p* < 0.05 or *p* < 0.001, Figure 1(c)). The above results suggested that PD showed a good hemostasis function on the mouse after subcutaneous injection for 4 h.

PD was injected subcutaneously into rats, which could be absorbed into the blood rapidly and affected the hemocyte parameters participating in the hemostasis process. PD mainly influenced the parameters of red blood cell (RBC, Figures 1(d)–1(f)) and platelet (PLT, Figures 1(g)–1(i)) of rats after subcutaneous injection for 4 h; however, other parameters (white blood cell counts, hemoglobin, neutrophils, lymphocyte, etc.) in blood routine had no changes (*p* > 0.05). 4 mg/kg PD increased slightly the RBC of rats, but it was not statistically significant compared with the NS group (Figure 1(d), *p* > 0.05). Red cell distribution width-standard deviation (RDW-SD, Figure 1(e)) and red cell distribution width-coefficient of variation (RDW-CV, Figure 1(f)) of rats treated with 2 mg/kg and 4 mg/kg PD were notably higher than these of NS group (*p* < 0.05 or *p* < 0.01). Importantly, compared with the NS group, PD obviously elevated PLT parameters including PLT counts (Figure 1(g)), plateletcrit (PCT, Figure 1(h)), and platelet larger cell ratio (P-LCR, Figure 1(i)) of rats (*p* < 0.05 or *p* < 0.01). These results showed that PD mainly affected the parameters of RBC and PLT of rats after 4 h treatment.

### 3.2. PD Affected APTT, TT, and FIB of Coagulation Parameters in Rat

**Figure 2:**
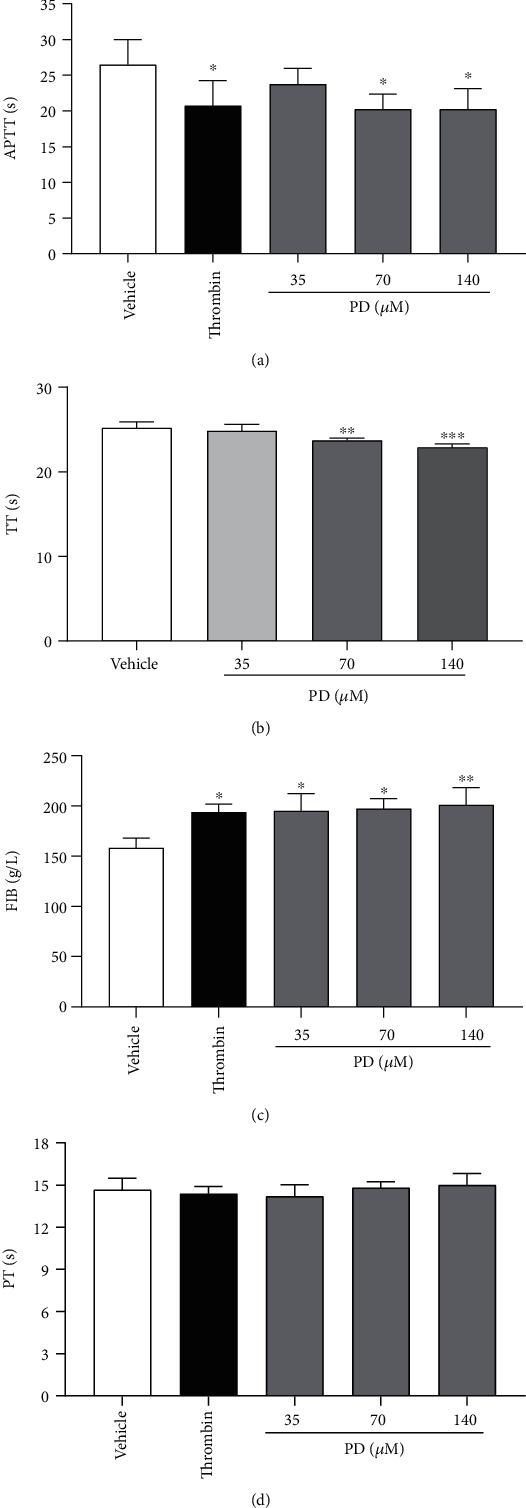
The effect of PD on coagulation parameters of rat plasma *in vitro*. (a) APTT. (b) TT. (c) FIB. (d) PT. The data are expressed as mean ± SD (*n* = 3). ^∗^*p* < 0.05 and ^∗∗^*p* < 0.01 compared to the vehicle group.

Based on the above results *in vivo*, PD showed hemostasis in rats, further research on the effect of PD on coagulation parameters. As compared to the vehicle group, the clotting time was significantly shortened by PD (70 and 140 *μ*M) in the APTT and TT assay (Figure 2(a), *p* < 0.05; Figure 2(b), *p* < 0.01 or *p* < 0.001); FIB concentration markedly increased in a dose-dependent manner (Figure 2(c), *p* < 0.05 or *p* < 0.01). However, PT had no difference between the vehicle group and PD treatment groups (Figure 2(d), *p* > 0.05). APTT reflects the endogenous coagulation system [32]; TT reflects anticoagulant and fibrinolytic substance in the common pathway of coagulation process that fibrinogen converted to fibrin [33], and FIB is an acute-phase protein in the last step of hemostasis [34]. So, the above results suggested that PD might play a hemostatic role by affecting APTT, TT, and FIB.

### 3.3. PD Activated Platelets by Promoting Calcium Influx, Releasing Granules, and Increasing GP IIb/IIIa Expression

**Figure 3:**
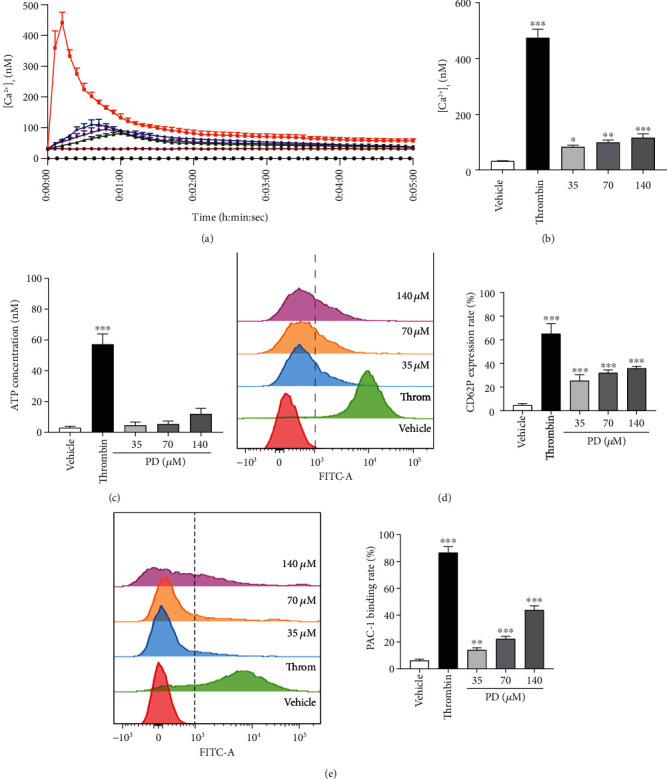
PD activated platelets without additional Ca^2+^. (a) The calcium kinetic curves of the human platelets. (b) The effect of PD on [Ca^2+^]*_i_* of human platelets at peak. (c) The effect of PD on ATP release of platelets. (d) The effect of PD on P-selectin (CD62P) of human washed platelets. (e) The effect of PD on PAC-1 of human washed platelets. The data are expressed as mean ± SD (*n* = 3). ^∗^*p* < 0.05, ^∗∗^*p* < 0.01, and ^∗∗∗^*p* < 0.001 compared to the vehicle group.

Platelets play an important role in primary hemostasis and wound healing [35]; intracellular Ca^2+^ mobilization participated in platelet activation, shape change, granule release, and aggregation [36]. PD on the human platelets calcium kinetic curve was detected by applying a cell imaging multimode reader.  Figure 3(a) shows that PD could only moderately increase Ca^2+^ concentration in human platelets over time; however, thrombin markedly instantaneously increased the Ca^2+^ influx into platelets.  Figure 3(b) shows that PD could significantly increase intracellular Ca^2+^ at peak in a dose-dependent manner (*p* < 0.05). Platelets are stimulated by agonists to release granules including a-granules and dense granules that further activate platelets. ATP (dense granule) release and P-selection (CD62P, a-granule) expression are commonly used as a marker to quantify the level of platelet activation [37].  Figure 3(c) shows that PD promoted the ATP release of human platelet, but it had no statistically significant compared with the vehicle group (*p* > 0.05).  Figure 3(d) shows that PD significantly increased the CD62P expression rate compared with the vehicle group (*p* < 0.05). Fibrinogen binding to GP IIb/IIIa receptor results in platelet aggregation [38]. GP IIb/IIIa on the platelet surface was detected by flow cytometry with antibody PAC-1 [39] that was studied to explore platelet aggregation further. As Figure 3(e) shows, the PAC-1 binding rate of human washed platelets in the PD group was significantly higher than that in the vehicle group (*p* < 0.001), and the max binding rate was 43.85% at 140 *μ*M. However, only weaker PD activates platelets by promoting Ca^2+^ influx, releasing granules, and increasing GP IIb/IIIa expression. Furthermore, we studied the effect of PD on human washed platelet with 1 mMCa^2+^.

### 3.4. PD Promoted Human/Rat-Washed Platelet Aggregation by Depending on Ca^2+^

**Figure 4:**
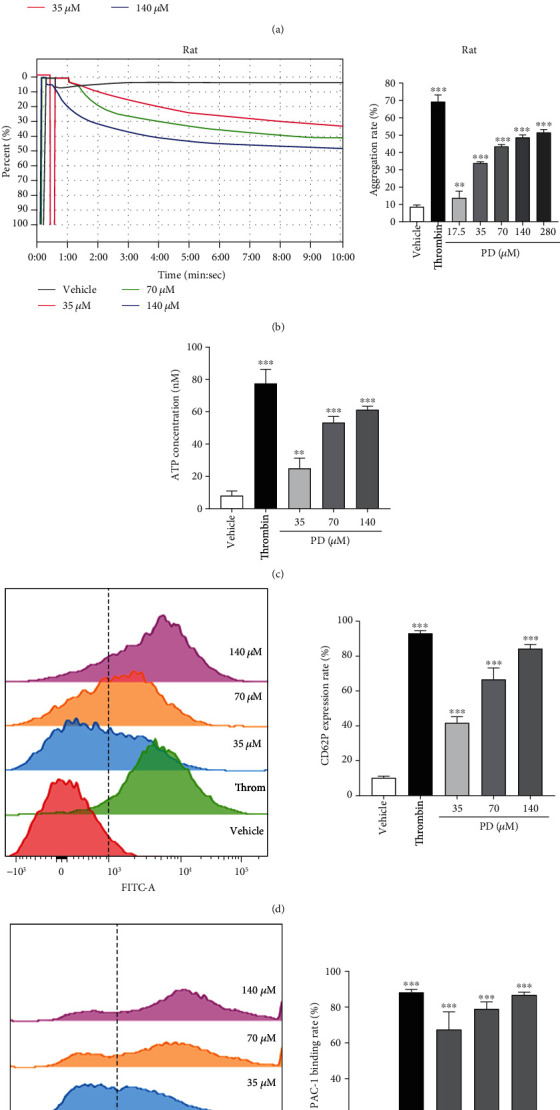
PD promoted human/rat-washed platelet aggregation by depending on high Ca^2+^ concentration. (a) The effect of PD on aggregation rate of human washed platelets with 1 mM Ca^2+^. (b) The effect of PD on aggregation rate of rat-washed platelets with 1 mM Ca^2^. (c) The effect of PD on the ATP release of human washed platelets with 1 mM Ca^2+^. (d) The effect of PD on the CD62P expression of human washed platelets with 1 mM Ca^2+^. (e) The effect of PD on the PAC-1 binding rate of human washed platelets with 1 mM Ca^2+^. The data are expressed as mean ± SD (*n* = 3). ^∗∗^*p* < 0.01 and ^∗∗∗^*p* < 0.001 compared to the vehicle group.

Figure 4(a) shows that the aggregation rate of human washed platelets with 1 mM Ca^2+^ treated with PD increased dramatically compared with the vehicle group in a dose-dependent manner (*p* < 0.001); the platelet aggregation rate was 36.00% at 280 *μ*M.  Figure 4(b) shows the same effect that PD induced rat-washed platelet aggregation. The rat platelet aggregation rate obviously elevated with the increase of PD concentration (*p* < 0.01 or *p* < 0.001); the max rat platelet aggregation rate was about 51.40% at 280 *μ*M. Obviously, the aggregation effect of PD on human/rat platelets including 1 mM Ca^2+^ was weaker than that of thrombin on human/rat platelets. The result indicated that PD could induce human/rat platelet aggregation; the aggregation effect of PD on human platelets was weaker than that on rat platelets.

After adding 1 mM Ca^2+^, PD dose-dependently increased ATP release of platelets (Figure 4(c), *p* < 0.01 or *p* < 0.001); however, ATP release of platelet without 1 mM Ca^2+^ was weaker than that of platelet with 1 mM Ca^2+^ (Figure 3(c)).  Figure 3(d) shows that PD promoted the CD62P expression rate of platelets; when the concentration of PD was 140 *μ*M, the CD62P expression rate was 35.80% (*p* < 0.001); after adding 1 mM Ca^2+^, PD significantly increased the CD62P expression rate of human platelets compared with the vehicle group, and the expression rate was 84.32% at 140 *μ*M PD (Figure 4(d)). Similarly, PD significantly elevated the PAC-1 binding rate of human platelets with 1 mM Ca^2+^, and the PAC-1 binding rate was 86.87% at 140 *μ*M, which was similar to that of thrombin (88.24%) on platelets (Figure 4(e)). The above results showed that adding large amounts of Ca^2+^ enhanced the activation of PD on platelets including granule release and GP IIb/IIIa expression to accelerate the platelets aggregation.

### 3.5. VP Inhibited PD-Induced Human Platelet Activation, Release, and Aggregation

**Figure 5:**
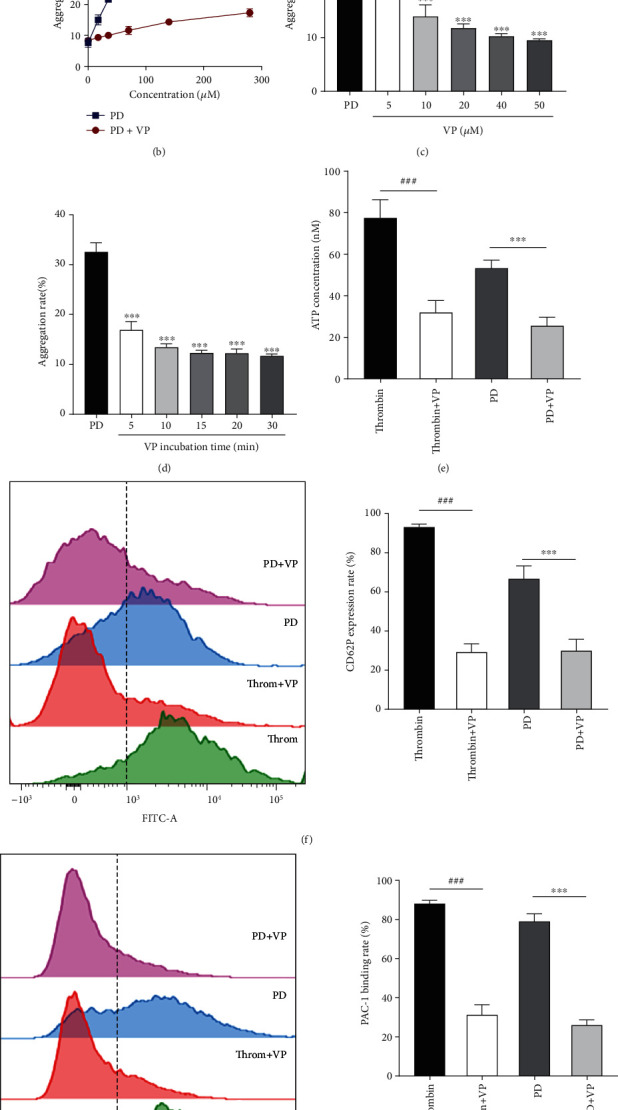
The effect of three inhibitors on PD-induced the human washed platelets aggregation. (a) The aggregation rate of PD alone or combined with VP, TG, or ST on human platelets. (b) VP inhibited the aggregation rate of platelets treated with the different concentrations of PD (140 *μ*M). (c) The different concentrations of VP inhibited the aggregation rate of platelets treated with PD. (d) VP at different preincubation times inhibited the aggregation rate of platelets treated with PD (140 *μ*M). (e) VP inhibited the ATP release of platelets treated with PD (140 *μ*M). (f) VP inhibited the CD62P expression rate of platelets treated with PD (140 *μ*M). (g) VP inhibited the PAC-1 binding rate of platelets treated with PD (140 *μ*M). (h) VP inhibited the [Ca^2+^]*_i_* of platelets treated with PD. (i) PD promoted the PAR1 expression of the human platelet. Vorapaxar: VP; ticagrelor: TG; seratrodast: ST. The data are expressed as mean ± SD (*n* = 3). ^∗^*p* < 0.05 and ^∗∗∗^*p* < 0.001 compared to the PD group; ^###^*p* < 0.001 compared to the thrombin group; ^&&^*p* < 0.01 and ^&&&^*p* < 0.001 compared to the vehicle group. VP: vorapaxar; TG: ticagrelor; ST: seratrodast.

Some receptors are expressed on activated platelets; thrombin receptors (PARs), ADP receptors (P_2_Y_12_, P2Y1, and P2X1), collagen receptors (GPVI and a2*β*1), and thromboxane A2 receptor (TP) are common. Therefore, we added receptor inhibitors to screen potential sites of action.  Figure 5(a) shows VP obviously inhibited PD-induced platelet aggregation (*p* < 0.001), and TG and ST did not change PD-induced platelet aggregation (*p* > 0.05). VP is the antagonist of the thrombin receptor (PAR1) [26]. Our results showed that PAR1 might participate in PD-induced human platelet aggregation. Thus, we further studied the effect of VP concentration, VP incubation time, and PD concentration on platelet aggregation.  Figure 5(b) shows that VP significantly inhibited PD-induced platelet aggregation; when the concentration of PD was 140 *μ*M, the inhibition rate of VP was 44.78% on human platelet aggregation. The aggregation rate reduced with the increase of VP concentration (5-50 *μ*M) and incubation time during 5-30 min (Figures 5(c) and 5(d)).

In a further development, we detected ATP release, CD62P expression, GP IIb/IIIa expression, and [Ca^2+^]*_i_* of PD combined VP on human platelets. The results showed that VP obviously inhibited increase of ATP (Figure 5(e)), CD62P (Figure 5(f)), PAC-1 (Figure 5(g)), and calcium influx (Figure 5(h)) of platelets induced by PD (*p* < 0.001). These results showed that PAR1 participated in PD-induced platelet activation, release, and aggregation. Therefore, we measured the PAR1 expression on human platelets induced by PD. As shown in Figure 5(i), PD markedly increased the PAR1 expression of human platelets (*p* < 0.01 and *p* < 0.01).

### 3.6. PD Affected the Downstream Signaling Targets of PAR1

**Figure 6:**
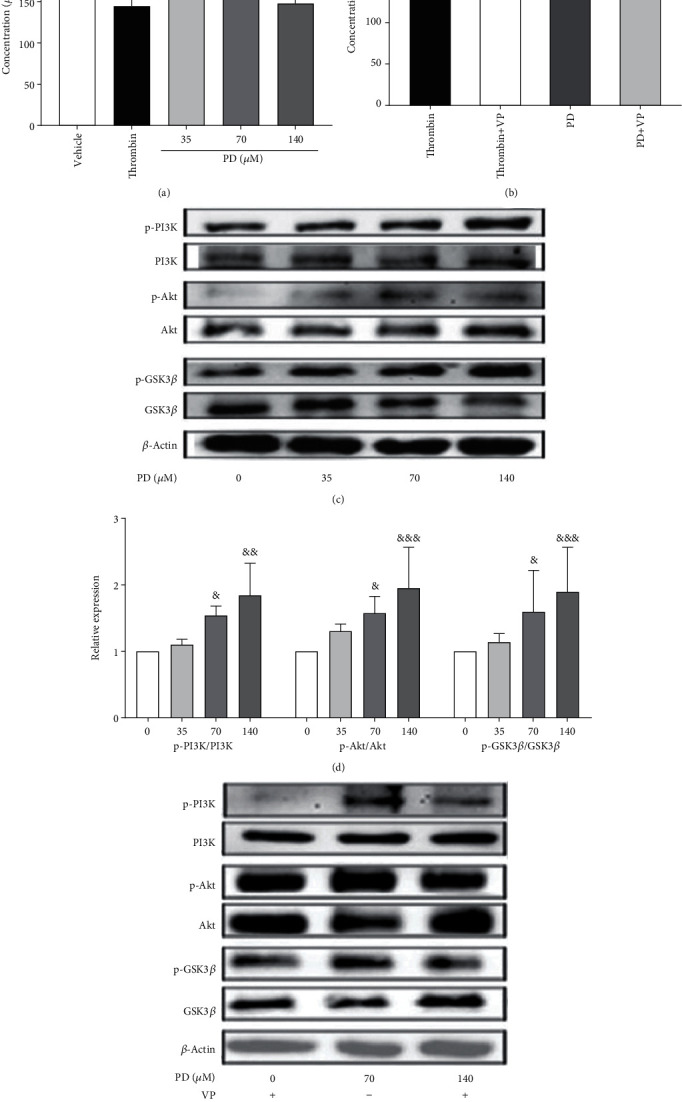
The effects of PD on downstream signaling targets of PAR1. (a) The effect of PD on the cAMP concentration of human washed platelets. (b) VP increased the cAMP content of human platelets treated with PD. (c) The expression of the protein by Western blot. (d) The effect of PD on phosphorylation levels of PI3K, Akt, and GSK3*β* in human platelets. (e) VP inhibited the phosphorylation levels of PI3K, Akt, and GSK3*β* of platelets treated with PD. The data are expressed as mean ± SD (*n* = 3). ^∗^*p* < 0.05 compared to the PD group; ^##^*p* < 0.01 compared to the thrombin group; ^&^*p* < 0.05, ^&&^*p* < 0.01, and ^&&&^*p* < 0.001 compared to the vehicle group.

PAR1 couples to G protein resulting in cAMP reduction and activation of the PI3K signaling pathway that plays an important role in platelet activation [40–42]. Therefore, we determined the cAMP level and phosphorylation of downstream intracellular signaling molecules including PI3K, Akt, and GSK3*β* in human platelet.  Figure 6(a) shows that PD could significantly inhibit the cAMP production of platelets compared with the vehicle group (*p* < 0.05, *p* < 0.01, or *p* < 0.001). After adding the VP, the reduction of cAMP concentration of platelets induced by PD was reversed (Figure 6(b), *p* < 0.05). Furthermore, Figures 6(c) and 6(d) show that PD markedly increased the phosphorylation levels of PI3K, Akt, and GSK3*β*; meanwhile, VP inhibited the phosphorylation levels of PI3K, Akt, and GSK3*β* of platelets induced by PD (Figure 6(e)). These results indicated that PD might activate the downstream proteins of PAR1, such as cAMP, PI3K, Akt, and GSK3*β*, to promote platelet aggregation; and VP inhibited the progress of platelet aggregation.

## 4. Discussion

Many components participate in the hemostasis process after vessel wall damage including plasma components, vascular components, and coagulation factors [17]. In this study, we evaluated the hemostatic effects of PD on human/rat platelets and coagulation parameters in *in vivo* and *in vitro* experiments.

First, bleeding models were used to evaluate the hemostatic effect of PD *in vivo*. We found that PD significantly shortened the bleeding time of mice with tail amputation and liver scratch (Figures 1(b) and 1(c)). Second, PD affected the routine blood parameters of rats after subcutaneous injection for 4 h. The results suggested that PD elevated significantly the RBC and PLT parameters (Figures 1(d)–1(i)). In the RBC parameters, increases of RBC-SD and RBC-CV reflect the changes in the heteronormocytic population, which often appear in cardiovascular disease [43]. Platelet plays an essential role in hemostasis and thrombosis under physiological or pathological conditions [19]. PCT provides more comprehensive data about total platelet mass [44]; high HCT values are associated with coronary artery disease [45]. P-LCR is the measure value of larger platelets; some reports have shown that large platelets are biologically more active and their prothrombotic properties are more powerful [46]. An increase in platelet counts, PCT, and P-LCR indicates the increase in platelet reactivity that has a prothrombotic tendency [47]. PD obviously increased RBC-SD, RBC-CV, PLT counts, HCT, and P-LCR of plasma in rats. These results suggested that PD influenced RBC and PLT parameters to promote hematopoiesis and thrombopoiesis [48]. Third, PD influenced the coagulation parameters *in vitro* by further study. Common coagulation parameters include the APTT, PT, TT, and FIB. Our results showed that PD remarkably decreased the APTT and PT and increased the FIB concentration of rats (Figures 2(a) and 2(b)). APTT is used to screen experiments for intrinsic coagulation pathways [32], which reflects the levels of coagulation factors VIII, IX, X, XI, and XII in plasma. TT is a simple and convenient test for detecting functions of coagulation, anticoagulation, and a fibrinolytic system, which reflects the conversion of fibrinogen to fibrin after addition of thrombin reagent [33, 49]. Soluble FIB is converted into insoluble fibrin that participates in hemostasis processes [34]. The fibrin and TT can evaluate inborn (congenital) and acquired qualitative and quantitative disorders of fibrinogen that can lead to thrombotic or bleeding events [49]. These results indicated that PD took part in hemostasis by affecting RBC and PLT, APTT, TT, and FIB.

Platelet participates in the hemostasis process including platelet counts and platelet aggregation. PD increased the platelet counts of rats *in vivo* that had been testified. At last, the process of PD on platelet *in vitro* further was studied. Under some agonists (ADP, thrombin, collagen, etc.) stimulating, the platelets in the blood vessel are activated to release a-granules (vWF, P-selectin, PF4, thrombospondin-1, etc.) and dense granules (ADP, ATP, 5-HT, histamine, etc.) [37]. Some granules such as ATP, ADP, 5-HT, and vWF activate circulating platelets to accelerate the platelet aggregates. Additionally, GP IIb/IIIa (PAC-1, as GP IIb/IIIa specific antibody) on activated platelets mediates platelet aggregation by binding adhesive proteins, which converts fibrinogen to insoluble fibrin [50]. In this study, PD increased ATP and P-selectin release of human platelets and elevated the PAC-1 binding rate of activated platelets (Figure 3). However, PD should be weaker agonists that still did not promote the washed platelet aggregation without 1 mM Ca^2+^.

Ca^2+^ signaling takes part in the process of platelet activation, shape change, granule release, thrombus formation, and GP IIb/IIIa expression [36, 51]. After adding 1 mM Ca^2+^, granule release (ATP and P-selectin) and GP IIb/IIIa expression of platelets treated with PD were significantly increased (Figure 4). The above results suggested that a large number of Ca^2+^ strengthened the effect of PD on washed platelets and collectively promoted platelet aggregation (Figures 4(a) and 4(b)). Ca^2+^ signaling plays a role in PD-induced platelet aggregation.

Many receptors such as GPIb/V/IX, GPVI, a2*β*1, PARs, P2Y_1_, P2Y_12_, TP, and integrins on activated platelet membrane are expressed [52]. VP, TG, and ST are antiplatelet drugs through antagonism of PAR1, P2Y12, and TP, respectively [53, 54], which are identified potential target sites of PD action. Our results showed that VP inhibited the platelet aggregation by inducing PD (Figure 5). VP, an antiplatelet agent, is an orally active, nonprotein, highly selective, competitive thrombin receptor (PAR1) inhibitor. PAR1 of human platelet participates in thrombin mediating platelet responses [22]. PAR1 activates heterotrimeric G proteins including the G12/13, Gq, and Gi/z family members [55] to impact the coagulation-related network of signaling pathways [24]. The *α*-subunits of G13 bind rho guanine nucleotide exchange factors (RhoGEFs) to participate in the shape change of platelets [56]. G*α*_q_ activates PLC*β* that increases intracellular Ca^2+^ and activation of protein kinase C [57]. These provide a pathway to calcium-dependent kinases and phosphatases, RhoGEFs, mitogen-activated protein kinases, and other proteins that mediate cellular responses ranging from granule secretion, integrin activation, and aggregation in platelets [24, 58]. PAR1 couples to G*α*_i_ subunit result in the G*βγ*-mediated activation of PI3K [41]. Both PLC*β* and PI3K mediate secretion, calcium response, and aggregation in platelets, which play vital roles in platelet cytoskeletal dynamics [59]. And G*α*_i_-mediated inhibition of adenylate cyclase reduces the release of cAMP in platelets [40]. In this study, PD had a similar active site on platelet with thrombin, it bounds to PAR1 of platelets to induce platelet aggregation, and the process was inhibited by VP (Figures 5(a) and 5(b)). Meanwhile, VP inhibited the release of ATP and P-selection, and GP IIb/IIIa expression of platelets stimulated by PD (Figures 5(c)–5(e)), which increased the level of cAMP to return to normal level (Figure 6(b)). The PI3K/Akt/GSK3*β* pathway is the major signaling axis regulating platelet aggregation [60]; PKC and Akt modulate platelet function by phosphorylating and inhibiting GSK3*α*/*β* on thrombin-mediated platelet activation [61]. Our results had shown that PD increased the phosphorylation level of PI3K, Akt, and GSK3*β* (Figures 6(c) and 6(d)); meanwhile, VP inhibited the phosphorylation levels of three proteins. These results suggested that PD activated the platelets by affecting PAR1 pathway.

In conclusion, PD, an aglycone of protopanaxadiol-type ginsenosides, had a hemostatic effect by promoting platelet aggregation and affecting the WBC, PLT, APTT, TT, and FIB. The possible mechanism of PD-induced platelet aggregation depended on calcium signaling and triggered platelet responses *via* the PAR1 and PI3K/Akt/GSK3*β* signaling pathway. PD, as the hemostatic ingredient, is an important discovery in *Panax notoginseng*, which will treat hemorrhage diseases in clinical.

## Data Availability

The data used to support the findings of this study are available from the corresponding authors upon request.

## Abbreviations

PD: 20(*S*)-Panaxadiol
ATP: Adenosine triphosphate
cAMP: Cyclic adenosine monophosphate
PT: Prothrombin
APTT: Activated partial thromboplastin time
TT: Thrombin time
FIB: Fibrinogen
HC: Hemocoagulase
PRP: Platelet-rich plasma
VP: Vorapaxar
TG: Ticagrelor
ST: Seratrodast
GP: Glycoprotein
PAR1: Protease-activated receptor 1
RBC: Red blood cell
PLT: Platelet
RDW-SD: Red cell distribution width-standard deviation
RDW-CV: Red cell distribution width-coefficient of variation
PCT: Plateletcrit
P-LCR: Platelet larger cell ratio

## Additional Points

Note: A preprint has previously been published [62].
